# African Local Pig Genetic Resources in the Context of Climate Change Adaptation

**DOI:** 10.3390/ani14162407

**Published:** 2024-08-19

**Authors:** Lenox Pius, Shuntao Huang, George Wanjala, Zoltán Bagi, Szilvia Kusza

**Affiliations:** 1 College of Animal Science and Technology, Huazhong Agricultural University, Wuhan 430070, China; piuslenox@yahoo.com (L.P.); shuntaohuang@webmail.hzau.edu.cn (S.H.); 2 Animal Breeding and Genetics Resource Section, Tanzania Livestock Research Institute (TALIRI), Dodoma 41207, Tanzania; 3 Centre for Agricultural Genomics and Biotechnology, University of Debrecen, Egyetem tér 1, 4032 Debrecen, Hungary; geog.wanjala@agr.unideb.hu (G.W.); bagiz@agr.unideb.hu (Z.B.); 4 Doctoral School of Animal Science, University of Debrecen, Böszörményi út 138, 4032 Debrecen, Hungary; 5 Institute of Animal Sciences and Wildlife Management, University of Szeged, Andrássy út 15, 6800 Hódmezővásárhely, Hungary

**Keywords:** African local pig genetic resource, diversity, natural selection, selection signature, microsatellite maker, production system, SNP genotyping

## Abstract

**Simple Summary:**

Pig farming is one of the most profitable components of the livestock sector in agriculture, significantly contributing to economic development, food security, and improved livelihoods for local communities in Africa and globally. However, with the increasing concern for global environmental changes, pig production is considered one of the vulnerable livestock sectors likely to be affected the most. Africa, being a tropical continent with extraordinary geographical and biological diversity, is believed to have varieties of local pigs exhibiting valuable genetic traits that can be used to promote livestock productivity through breeding for climate-resilient breeds. Unfortunately, many of these valuable traits have not been fully identified and exploited. This study provides an overview of the current state of African pig genetic resources by highlighting their diversity and adaptability potential from both phenotypic and genetic evidence. Our results indicate that African local pigs hold potential genetic traits critical for climate change adaptation. However, these traits are threatened due to crossbreeding activities with commercial breeds that are now prevalent across the continent. Thus, to keep up with the rapid speed of climate change, efforts to realize and utilize these considerable potential traits must increase before they are permanently depleted.

**Abstract:**

Africa is home to a wide diversity of locally adapted pig breeds whose genetic architecture offers important insights into livestock adaptation to climate change. However, the majority of these inherent traits have not been fully highlighted. This review presents an overview of the current state of African pig genetic resources, providing highlights on their population and production statistics, production system, population diversity indices, and genomic evidence underlying their evolutionary potential. The study results reveal an incomplete characterization of local pig genotypes across the continent. The characterized population, however, demonstrates moderate to high levels of genetic diversity, enough to support breeding and conservation programs. Owing to low genetic differentiation and limited evidence of distinct population structures, it appears that most local pig populations are strains within larger breeds. Genomic evidence has shown a higher number of selection signatures associated with various economically important traits, thus making them potential candidates for climate change adaptation. The reportedly early evidence of hybridization with wild suid groups further suggests untapped insights into disease resistance and resilience traits that need to be illuminated using higher-density markers. Nevertheless, gene introgression from commercial breeds is prevalent across Africa; thus, efforts to realize and utilize these traits must increase before they are permanently depleted.

## 1. Introduction

Pig farming is one of the most profitable components of the livestock sector in agriculture, proven to contribute to economic development, food security, and improved livelihoods for local communities around the world [1,2]. According to the recent statistics released by FAO in 2022, pork is the second most widely consumed meat globally, accounting for 36% of total meat consumption, behind poultry at 40% and ahead of beef at 22% [2]. Similarly, in Africa, despite religious constraints that have historically limited pig farming in some regions, pork consumption is also ranked second after poultry [1]. With the growing global human population and economic development trends, the demand for animal protein, pork in particular, is expected to rise significantly at the household level, and at the same time, competition for resources (feed, water, land, etc.) will intensify as environmental changes continue to transform, thus requiring an increased supply of pork [2,3]. For instance, predictive models suggest that livestock production in developing countries may undergo a reduction of 12–30% by 2050 due to climate change [4]. This implies that livestock production systems need to become more productive and efficient to meet the increasing demand, along with the anticipated climate change effect [1,3,5]. In recent years, following advances in molecular biology technologies, there has been increasing recognition that the diversity of farm animal genetic resources can play an important role as a strategy for adaptation to climate change [6,7]. Livestock breeds have varying levels of resilience to stresses caused by climate change, and thus, breeds that are well adapted have a better chance of sustaining livelihoods and food security despite the anticipated changes in environmental conditions [6,7].

Africa is known for having a wide variety of local pigs that have genetically adapted to survive in challenging environments, such as high temperatures, illnesses, and limited access to food [8,9]. These genetic resources present an opportunity to select climate-resilient animals within tolerant breeds or identify resistance genes for incorporation into commercial lines or other strains [10,11]. Nevertheless, as a result of their low productivity, these breeds are disregarded in favor of their superior counterparts, leading to their vulnerability to extinction. It is important to mention that these superior breeds have been intensely selected for lean growth and fertility traits, and they are thereby more susceptible to heat stress and other environmental challenges [6,7]. Thus to achieve sustainable management of the pig industry alongside the anticipated climate challenges, a holistic approach should be undertaken to include local pig genotypes in breeding programs as a means of imparting their adaptive characteristics [10,12].

While local pig genotypes have been recognized as key players in the continental agricultural and cultural landscape for many years, there is still sparse and unreliable genetic information across many countries in Africa [9,10,13]. Studies have analyzed and reported the genetic and phenotypic characteristics of certain pig breeds in separate African nations, but this has restricted our knowledge of the general status of the African pig sector [9,14,15]. Therefore, this review article aims to enhance our understanding and promote the exploitation of genomic architecture underlying some important economic traits reported among African local pig genetic resources. It provides an overview of the present status, focusing on population and production trends, morphological and phenotypic characteristics, population genomics, and, most importantly, signatures of selection underlying some important alleles associated with traits of economic importance among local pig populations across the continent.

## 2. Materials and Methods

This scoping literature review aims to examine the status and evolutionary potential of some African local pig genetic resources in the context of climate change adaptation. The literature included in this study was sourced from academic databases such as Scholar, Web of Science, Springer, Wiley Online Library, ProQuest SciTech Collection, and Science Direct. Keywords including pig genetic resource, production system, population structure, selection signature, microsatellite maker, gene sequencing, and SNP genotyping, combined with local pigs and Africa, were employed as search criteria. These keywords were also combined with the appropriate country name to facilitate the filtering of the search results. Relevant publications were identified through a careful examination of the titles, abstracts, and complete texts. 

## 3. Results 

### 3.1. Population Distribution and Production Status of the Pig Sector in Africa

**Figure 1:**
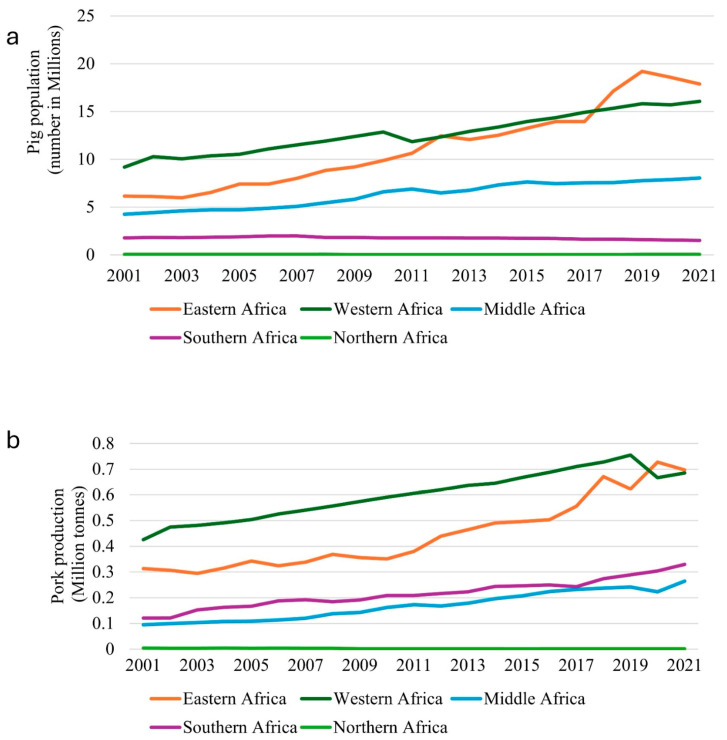
Trends in pig population and pork production across African regions from 2001 to 2021. (**a**) Pig population (in millions) by region, showing the highest populations in Eastern and Southern Africa (**b**) Pork production (in million tonnes) by region, with Eastern Africa leading in production, followed by Southern Africa [18].

**Figure 2:**
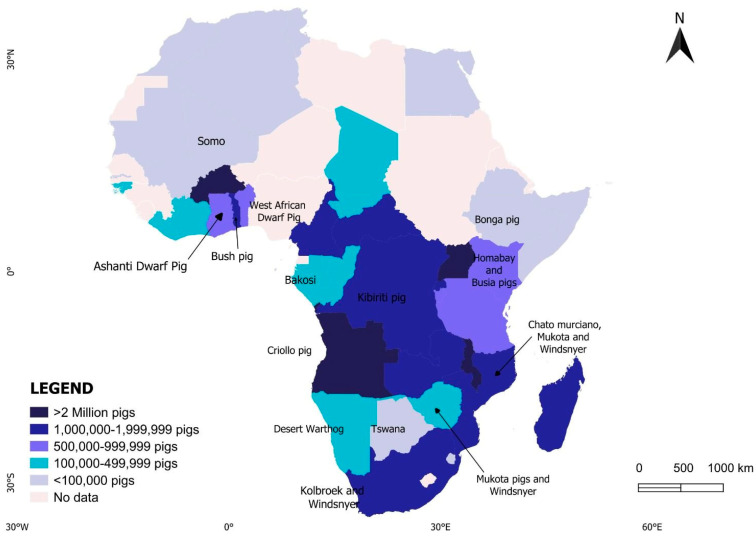
Pig population distribution in different countries across the continent in 2021 [18].

In many African countries where pigs are culturally accepted, pig farming presents an essential component of food and nutritional security, as well as income generation, to many rural farmers in Africa, and the same applies to many other developing areas [16,17]. The pig industry in Africa has experienced significant growth over the past two decades [1]. According to FAOSTAT, the pig population in Africa has increased dramatically from approximately 6 million heads in 2001 to over 43.5 million heads in 2021 (Figure 1). Concurrently, pork production has doubled, rising from less than one million metric tons (0.95 million metric tons) in 2001 to about 2.1 million metric tons in 2021 (FAO, 2022). This substantial growth reflects the evolving landscape of livestock farming and meat consumption, driven by changing economic conditions, population growth, and urbanization which are all contributing to an increase in societal demands. For many years, the pig population in Africa has not been uniformly distributed; instead, it is highly concentrated in Sub-Saharan Africa (SSA). For instance, of the 43.5 million pigs recorded in 2021, the highest regional distribution was recorded in eastern (17.7 million), western (16.1 million), central (8 million), southern (1.5 million), and northern Africa (0.39 million), respectively [18]. These estimated figures account for over 4.6% of the total world pig population (978.7 million) [1]. The pig population in northern Africa is extremely low due to religious beliefs and cultural traditions prohibiting pork consumption. According to FAOSTART 2021 estimate figures, Nigeria (8 million), Malawi (7 million), Angola (3.65 million), Burkina Faso (2.7 million), Uganda (2.6 million), Mozambique (1.6 million), and South Africa (1.34 million) are the top countries with the highest pig populations in the region [18]. The population distribution of pigs in different countries across the continent in 2021 is visually represented in Figure 2 [18]. 

On the other hand, 2.1 million metric tons of pork produced in 2021 contributed only to 1.67% of global pork production [1]. Among African countries, Nigeria and South Africa have been considered the top pork producers in the region with each producing around 300 thousand metric tons every year [18]. Other major producers in the top five include Malawi (279.9 thousand metric tons), Angola (136.4 thousand metric tons), Uganda (131.2 thousand metric tons), and Mozambique (121 thousand metric tons) [18].

### 3.2. Pig Production Systems in Africa

Generally, the pig production system in Africa extends from the commercially intensive sector to the traditional system (scavenging), in which pigs are reared by poorer farmers in rural areas [1,8]. The commercial production system often accommodates international commercial breeds such as Large White, Landrace, Yorkshire, Duroc, Hampshire, and Pietrain [1,8,16,17]. On the other hand, the village production system, which is the main focus of this study, is characterized by heterogeneous and non-described populations often raised under traditional low-input systems [8,9]. In between, there are semi-intensive systems characterized by smallholder groups of farmers with intermediary levels of management [1]. The dominant breeds in these systems are crossbreeds of commercial and local breeds though sometimes commercial breeds also exist [1]. Most pigs, accounting for 65–80%, are raised in traditional and semi-intensive systems (smallholders), though intensive production systems are also steadily increasing in peri-urban regions [1,16,19]. In addition to the local pigs found in this system, there are also several varieties of wild pigs native to the continent that roam freely near forests and wilderness areas, where they often freely interact with scavenging pigs [8]. Within traditional farming, smallholder pig farmers do not have formal breeding programs, nor do they practice the systematic selection of pigs. In this system, pigs are usually raised for subsistence, and farmers usually lack access to input like feed, veterinary services, and marketing-related information that may increase output [16,20]. Due to limited feed resources, pigs are allowed to roam freely in search of food, exposing them to extreme weather conditions, disease infections, and even theft [1]. This system is facing significant disease challenges, with African swine fever (ASF) being the most prevalent and economic one. Swine fever has been recognized to be endemic in Africa since when it was first formally identified in Kenya in 1921 [9]. For example, ASF caused the loss of about 25% of the global pig population between 2018 and 2019, leading to significant economic losses and hampering pig production in affected areas, thereby resulting in a decline in pig populations and impacting livelihoods [1]. Till recently, there had been no vaccines or any other proper treatment methods available for the treatment of ASF in Africa. Other common diseases of economic importance in rural areas are mange, respiratory infections, genital infections, foot rot, and swine diarrhea, particularly among piglets. Parasitic infestations such as worms, ticks, and lice are also widespread [21,22]. Weak veterinary services in rural areas, owing to poor infrastructure such as roads and electricity, significantly contribute to the rapid spread of preventable diseases [21,22,23]. Several studies from different countries have highlighted that many farmers within traditional free-ranging systems are unfamiliar with basic management practices such as vaccination, deworming, and dipping [21,22]. Some farmers even believe indigenous pigs are disease-resistant to the extent that they do not require veterinary services [21,24]. In many cases, farmers have been reported to use traditional methods or inappropriately use human medicines to treat animal diseases [21,22,25]. Engine oil, lime water, and ash–salt solutions have been largely reportedly used in the control of mange and other external parasites [21,24]. Therefore, due to longtime exposure to diseases and forces of natural selection, the majority of the pig population in this system is believed to have evolved unique genetic backgrounds conferring resilience against harsh conditions. 

### 3.3. Morphological Description, Production, and Reproduction Traits

Generally speaking, the diversity of the native pig population across Africa remains largely uncharacterized, leading to the existence of a variety of pigs with unknown genotypes. In 2014/15, African member states were asked to report on the characterization of AnGR Farm Animal Genetic Resources in their countries and the progress made over the past two decades. Out of 42 member states, 19 did not provide data on pigs [26]. Of those who responded, 14 conducted basic surveys, 3 performed quantitative studies, and only 5 carried out advanced characterization, highlighting a significant gap in characterization efforts [26]. For example, in countries like Tanzania, where the current first author is coming from, local pig genetic resources have never been genetically characterized to date. 

**Figure 3:**
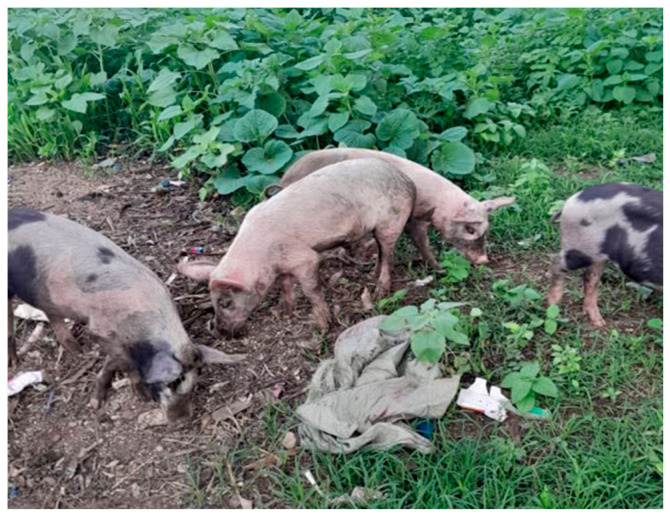
Free-ranging pigs of unknown genotypes in Tanzania.

Most of these breeds are native and display a broad spectrum of phenotypic characteristics [19,27]. Owing to their disadvantaged productivity, local pigs have continued to receive less attention compared to commercial breeds, resulting in their vulnerability to extinction [9,27]. Adding to the complexity, several local pig populations in Africa do not have official recognition as separate breeds. This leads to local pig populations with unknown genetic backgrounds [19,27] (Figure 3). Except for a few cases, like in South Africa, where there are some standard breeds native to the country (Windsnyer and Kolbroek), the genetic identity of most local pig breeds has been largely speculative, with sparse and often generalized phenotypic descriptions found in the academic literature [8,9]. The lack of defined genotypes leaves local pig populations across Africa identified by a confusing array of regional names, including Somo in Mali, West African Dwarf Pig in Nigeria, Ashanti Dwarf Pig in Ghana, Bush Pig in Togo, and others like Bakosi (Gabon), Busia (Kenya), Kibiriti (DR Congo), Bonga (Ethiopia), Desert Warthog (Namibia), Criollo (Angola), Chato Murciano (Mozambique), Tswana (Botswana), Mukota (Zimbabwe), Kolbroek (South Africa), and many more diverse terms [26]. The few studies that have performed phenotypic characterizations of local pigs in their production system have included [28,29,30] Eastern Africa [17,31,32,33], West Africa [34,35,36], and Southern Africa, among many others. Owing to their comparable morphology, local African pigs have been characterized as small framed groups of suid with long legs suited for roaming and scavenging [17,37]. Typical African local pig genotypes are usually slow growers with small-framed bodies (40–50 cm wither height), and they may rarely weigh above 60 kg as adults, even under optimum rearing conditions [29,32,38]. These values are greatly inferior compared to their improved counterparts with a record of a wither height of more than 75.1 m and a mature body over three times larger under optimum conditions [39] Phenotypically, they usually have a short forehead, elongated to medium snout, straight tails, and small-sized upright or slightly erected ears [29,32,38]. Unlike commercial breeds, which are over 79.1 cm long with bent backs, the majority of locals are comparatively short (52.2 cm long) with relatively straight backs [39]. Their skin and coat color varies from black to spotted, gray, red, or rarely white. The coat can be short or long with a mane down the back [29,32,38]. Despite their relatively disadvantaged body size, local pigs may have relatively good average dressed carcasses (70–75 percent), and the literature has indicated a high potential for improving their growth rate in market-oriented production systems through crossbreeding with exotic breeds for market-oriented systems [40,41]. Compared to commercial breeds, local pigs exhibit lower reproductive performance even with improved management. Local pigs have been reported to exhibit relatively smaller litter sizes at birth, ranging from 5.1 to 6.3 piglets, compared to 6.8 to 9.86 piglets in commercial breeds such as Large White and their crosses [42,43,44] Additionally, there is a noticeable disparity in birth weights with commercial breed records ranging from 1.3 kg to 1.95 kg, while average figures for local pigs range between 0.8 kg and 1.55 kg [43,44]. Weaning weights also differ markedly, with local pigs weighing as low as 3.8 kg compared to the average weight of 8 kg in commercial breeds [42,43,44]. While local breeds might seemingly have poor performance, their increasing utility in rural areas relies on the inherent adaptive trait that makes them thrive better in low-input systems, such as their ability to withstand food scarcity, tolerate heat stress, and resist diseases and parasites [45,46,47]. These traits allow them to have relatively stable production levels even in challenging environments where high-producing animals cannot accede. These resilient traits have contributed to their value and utility for smallholder village farmers for many years [9,35,48]. These adaptability traits that form the background of this review are discussed in detail in the next section. Beyond their resilient traits, local pig breeds hold significant cultural and historical importance since some breeds are closely tied to local identities and specific cultural practices [49,50,51,52]

Table 1 summarizes the morphological description, production, and reproduction performance of some African local pigs in various African countries.

### 3.4. Genetic Characterization of African Native Pig Breeds

Our literature search revealed that the microsatellite markers were the most commonly employed technique for genotyping local pig populations across the continent. In addition to microsatellite studies, four recent studies employed SNP arrays for genotyping providing more insight. Table 2 summarizes genetic diversity indices of local pig populations from 10 studies conducted in various countries across the continent.

The number of microsatellites utilized in these studies ranges from 12 to 39. Genetic diversity within and across populations is commonly measured using parameters such as the average number of alleles (Na), observed heterozygosity (Ho), and expected heterozygosity (He). The overall finding of these studies indicates that local pig populations exhibited moderate to high levels of genetic diversity compared to exotic breeds with some evidence of a lack of population substructure [9,15,36]. The mean observed and expected heterozygosity for microsatellite studies ranged from 0.41 in Nigeria pigs to 0.609 in South African pigs, whereas the expected heterozygosity (He) ranged from 0.46 in Nigeria to 0.723 in Ghana (Table 2). These values exceed those recorded on commercial breeds elsewhere [74]. The diversity indices are comparable with those for Mexican pigs [74], Iberian pigs [75], and Chinese pigs [76]. Ramírez et al. (2009) hypothesized that the moderate to high levels of heterozygosity observed to be exhibited by local breeds compared to their counterparts are attributed to the absence of selective breeding programs and the potential presence of diverse genetic lineages within these populations [77].

Besides their substantial diversity, local pig populations have been reported to exhibit low genetic differentiation between them, suggesting a lack of distinct substructures [15,36]. Though there are some exceptional cases in West Africa, the reported lower genetic differentiation can be demonstrated with lower observed heterozygosity (Ho) values compared to expected heterozygosity (He), as indicated in Table 2. Generally, when He exceeds Ho, it indicates the presence of population structure within the studied group, and the opposite is true [10,72]. Although there are some exceptional in West Africa where some distinct population structures have been reported, the incidence of lower levels of genetic differentiation among local pig populations has been extensively reported in the South African region [8,35,36]. In Africa, native pig populations are usually found in small, isolated flocks; thus, the lower levels of population differentiation may be attributed to the continuous gene flows between populations in nearby households that do not practice controlled breeding [8,10]. These findings provide credence to the notion that a large number of African local pig populations within and sometimes outside countries are strained within larger breeds [8,35,36]. This has breeding implications in reducing the necessity for separate conservation efforts for each strain and instead suggests pooling resources to manage and conserve local pigs in Africa effectively [8].

It is also important to acknowledge that these findings were carried out at different points in time, some of which were many years ago. Since that time, the genetic makeup of local pig breeds is believed to have undergone considerable dramatic social and environmental events that have led to a drastic decrease in their genetic diversity [13]. For instance, despite the higher genetic diversity previously reported for the Ghanian population [56], a recent genomic study utilizing a porcine 60K SNP array reported relatively lower genetic differentiation, indicating a higher degree of population relatedness (−0.09 to 0.43) [10]. Similarly in other countries, high-density genomic studies utilizing a porcine SNP array have continued to reveal diminishing levels of between- and within-population diversity in South Africa [8], Kenya [9], and Uganda [75], to mention a few. These studies have also exposed a significant level of admixture from commercial breeds (Table 2). Ho ranged from 0.23 in Kenya and 0.385 in South Africa, respectively, whereas the expected heterozygosity (He) ranged from 0.339 in South Africa to 0.88 in Busia pigs in Kenya (Table 2). Further, the inbreeding coefficients (F) in some African native pig breeds were alarmingly high, ranging from 0.043 to 0.198. The inbreeding levels recorded for southern African local pigs (0.198) and Ghanaian populations (0.15) were even two times greater than those recorded in Kenyan breeds (Busia, 0.07) and Homabay (0.09) pigs [9,10]. It is, however, important to note that previous research on these pig populations primarily used microsatellite markers, which are known for their high polymorphism. In contrast, more recent studies often use single nucleotide polymorphisms (SNPs), which are biallelic, meaning they typically have two alleles. Due to these fundamental differences in the genetic markers used, comparing these indices to account for the loss of diversity may not be appropriate [8]. Nevertheless, the observed high inbreeding level is alarming and may have severe negative impacts on genetic diversity, indicating a significant risk of extinction [8,10,73].

### 3.5. Adaptive/Resilient Traits of the Native Pig Population

The most highlighted and embraced comparative advantage of the local African pig genotypes are their hardiness in terms of disease and parasite tolerance, elevated thermos-tolerance, scavenging, and foraging ability, and good mothering ability, which make them more suited to low-input production environments [35,55,78]. Nevertheless, as highlighted earlier, the genomic information underlying these adaptive traits, which, of course, are ostensibly favored by smallholder farmers, is limited and mostly based on subjective evidence and local knowledge [9]. These positive characteristics of local pigs are valuable and, therefore, need to be characterized and conserved, as they are also important to the livelihood of small-scale farmers and women in particular [79]. We hereunder provide an in-depth description of the adaptive potential of these traits in local African pigs based on documented anecdotal evidence and previous quantitative research to get a fundamental overview. We thereafter end our discussion by presenting recent molecular evidence that has recently shed light on the genomic architecture associated with previously discussed adaptive traits in local pigs.

#### 3.5.1. Disease- and Parasite-Resilient Attributes

The local pigs are believed to exhibit significant resilience to diseases and parasites that would easily subdue exotic pig breeds [37,55,78]. For instance, studies have provided foundational pieces of evidence suggesting that local pigs have lower worm burdens and less liver damage from *Ascaris suum* compared to exotic breeds [68]. They are also considered potential reservoirs of gastrointestinal nematode resistance genes, while others are reported to have higher antibody responses to erysipelas, parasitism, and other endemic diseases [67,80,81]. We mention a few local breeds like Mukota pigs in Zimbabwe [81] and black hairy Nigerian Local pigs [82], and many other localized local strains have been reported to be tolerant to lethal African swine outbreaks, though the genetic basis underlying their tolerance to infection is yet not unclear. Likewise, a significant proportion of domestic pig populations from Kenya [83,84], Tanzania [85], Uganda [84], Congo [78], and Zambia [86] have been reported to remain asymptomatic despite harboring the ASF virus in their tissues thus, indicating some form of tolerance to infection. Recently, in Kenya, in an attempt to explore the host genetics that showed resistance to ASF, Mujibi et al. (2018) found that pigs with higher local ancestry exhibited resistance to ASF [9].

#### 3.5.2. Thermotolerance Attributes

Considerable variations exist between and within species in their ability to tolerate heat stress [87]. Local pig breeds are speculated to be heat stress-tolerant, a survival trait that allows them to thrive in hot tropical climates [88] where they are predominantly reared in outdoor environments [1]. The physiological adaptation of local breeds to heat stress could be partly explained by their lower production potential, which consequently contributes to less metabolic heat production [89,90]. In addition, their morphological features, such as small body sizes, longer snouts, and erect ears, aid in providing a thermoregulatory advantage, enabling more effective heat dissipation and increased reliance on evaporative cooling [5]. Behavioral adaptations such as wallowing behavior and frequency of drinking water daily are also important indicative features of heat stress tolerance in pigs [90]. For instance, the Mukota breed has been reported to have very low water consumption, likely due to favorable alleles related to heat tolerance [41]. Additionally, the predominant black color found in most local pigs is linked with heat tolerance and is reported to confer camouflage against predators during outdoor foraging [15]. Darfour-Oduro et al. claimed that Ashanti Black pigs native to Ghana are well adapted to tropical environments because they can withstand sunstroke [53]. Recently, a genomic study revealed an interesting finding regarding coat color variations in Dwarf pigs, highlighting some specific genes that were correlated in different ecological zones [10]. Additionally, some researchers argued that the mud found in the free-ranging systems also makes a significant contribution to preventing sunburn by coating the skin of the pig [46].

#### 3.5.3. Foraging and Scavenging Ability

Local pigs are also excellent foragers capable of subsisting on low-quality feeds, which reduces feeding costs for smallholder farmers [91]. Foraging ability enables free-range pigs to scavenge and survive on limited feed resources, reducing feeding costs for farmers and contributing to their utility for smallholder poor farmers [65]. Their long snouts, strong jaws, and heightened olfactory senses enhance their foraging abilities [65]. Anatomical adaptations like an enlarged caecum allow these pigs to thrive on high-fiber diets [66,92]. A digestibility and feeding trial was conducted to compare Mukota and Large White breeds, and they were cross-fed similar diets with increasing levels of maize cob meal. It established that Mukota pigs and their cross were able to utilize high-fiber diets better than the Large White pigs [65]. More intriguingly, the digestibility trial showed that, as the fiber content of the diet increased, Mukota pigs were better at using nutrients than Large White pigs. Local black Iberian pigs have a higher intake of acorns, grass, and roots while foraging in outdoor systems [91]. Recent genomic studies have started to uncover the genetic basis of these traits [8,13].

#### 3.5.4. Mothering Ability

Mothering ability greatly impacts pre-weaning mortality and overall piglet growth [93], and it is one of the traits of economic importance in the traditional production system, where piglets must be constantly protected from predators found in the free-ranging environment [94]. Local pig breeds generally display good mothering abilities [94]; however, only limited studies have fully examined the genetic basis and economic value of this trait [95]. Some breeds, like the Mukota of Zimbabwe [94], the Ashanti Dwarf of Ghana [48], and the Black pig of Nigeria, have been reported to exhibit better maternal behavior [82]. Chimonyo et al. reported that Mukota pigs possess genetically superior mothering abilities compared to commercial breeds, and they can notably raise piglets without farrowing crates [94]. While genetic studies on mothering abilities are also still limited, it is believed that outdoor rearing and natural social groups reduce stress, leading to calmer temperaments and better mothering behaviors [95].

### 3.6. Insights into Genetic Regions, SNPs, and Associated Genes Related to Various Traits in Local Pigs

Research on the genomes of African local pigs was limited for many years [10]. Nevertheless, recent research studies utilizing high density have continued to identify unique genomic regions under selection associated with crucial adaptive and economical traits in the local pig population, allowing researchers to positively use this data on their breeding programs [12]. A summary of genetic regions, SNPs, and associated genes related to various traits detected in various local pig populations in Angola [13], Kenya [9], Southern Africa [8,12], and Ghana [10] is presented in Table 3. These studies have revealed SNPs associated with quantitative loci (QTLs) related to economically important traits such as disease resilience and immune response (SWAP70, SBF2, ARHGAP23, ITFG2, and IL17B), climate resilience, and feeding efficiency (RPL18, DNAJC15, NPY2R, POLR3B, STAM2, DLGAP2, and ATPB2), growth-related traits (TSPAN9, DLX1, NKAIN3, ITGA2, and LIPA), meat quality-related traits (SEC13, JPH1, SCPEP1, NME1, FHL3, ARPC4, and SELENOP), reproduction and fertility (EPSTI1, ZNF609, MHR2, TARBP2, PIK3R5, FRAS1, and TCP11L2,), and fat deposition and metabolism (EPSTI1, SAMD4, IDE, DOCK5, NEDD4, and EXTL3), among many others (Table 1). Some of these selection signatures were found to be unique to specific local breeds, while others were identified in multiple comparisons between breeds [8,9,11,12,13]. It is, however, important to note that some of these studies were undertaken using density SNP arrays with limited resolution and low statistical methods, which may have affected the validity of certain results [9]. The following is an in-depth discussion of the breeding implications of some of the identified genes.

The first attempt to analyze genetic selection patterns in various South African pig populations applying advanced statistical methods (iHS, XP-EHH, and hapFLK) found numerous regions under significant selection in South African village pig populations compared to other populations [12]. In this study, the number of selection signatures in local pig populations was reportedly higher than in any other population, implying higher genetic diversity, which is likely to provide a broader pool of genetic variants for selection to act upon [12]. Signatures associated with immune response genes such as APBB2 (the regulation of inflammatory responses to porcine reproductive and respiratory syndrome virus) [125] and the LIPA gene, playing a role in wound response and inflammation [121], were among the important discoveries detected in village pigs, underscoring the genetic basis of the resilience traits of South African local pigs in the face of common pig diseases [12]. KCNJ3 was also identified in the Kolbroek and Windsyner breeds (improved breeds native to South Africa). The KCNJ3 gene is associated with signaling pathways that regulate fundamental functions such as temperature control and inflammation response [126], further highlighting the genetic basis of the resilience ability of the South African indigenous pig population. Further, a pairwise comparison of South African village pigs (Alfred NZO) and warthogs yielded resilient genes associated with both reproduction (RPL18 and IL17B) and growth traits (IL17B and ARHGAP23) [8]. IL17B and ARHGAP23 are immune-response genes linked to inflammatory responses, and they likely provide resilience in a scavenging environment [10]. A similar discovery of the IL7B gene, a crucial trait thought to be essential for food digestion and nutrient absorption in scavenging pigs, was also recently detected among the local pig population in Ghana, suggesting its important resilience role in rendering a protective role against adverse environmental conditions [10]. Furthermore, some potential reproductive traits, such as mothering ability and the hardiness of sows, which ensure high survival rates for litters, have also been illuminated in the South African pig population [12]. For instance, genes influencing litter size and piglet survival, such as PIK3R5, were identified in the South Village pig population [12]. Genomic regions containing QTLs associated with teat number, crucial for piglet survival and growth, were detected in the same population [12]. The author argued that, while commercial breeds may have higher litter sizes, extremely large litters can have negative implications for sow and piglet welfare. The need to use this gene in balancing reproductive traits could offer a more sustainable approach to pig production, particularly in low-input environments [12].

Contrary to what is believed by many, genes that are closely associated with meat quality traits were also identified in village pigs, underscoring the diversity of these South African local genotypes. For instance, a gene that regulates body fat content and correlates with intramuscular fat deposition, such as SCPEP and SAMD4A, was identified in South African village pig populations [12]. On the other hand, the BRPF1 gene, which is associated with intramuscular fat identified between warthogs and Windsnyer pigs (an improved indigenous breed), was considered an important discovery in this study [8]. The author argued that higher levels of intramuscular fat are associated with improved juiciness and tenderness in pork meat and are highly valued by consumers [8]. The NPY5R gene found in village and indigenous pig breeds is linked to feed efficiency and fat storage. This likely explains why local pigs, including Kolbroek, tend to store fat in their backs and bellies, an adaptation to survive periods of food shortage [8,60]. The same gene has been associated with fat deposition in local pig populations elsewhere in the world, such as in Chinese breeds [127].

The peak genes that were identified within the top 1% (ANKFN1, ARID1A, ATP8A2, and CNTN4) in the local Ghanaian population [10] were associated with abnormal eating behaviors, hyperactivity, decreased body weight, and decreased embryo size [122,123,124]. Chromosome 4 in Ashanti Dwarf pigs shows a high SNP concentration, linked to growth and reproduction. The IL7 gene is known to influence growth and reproduction, while it may also paradoxically reduce feed intake and growth [128], which was among the important discoveries [10]. Nine genes linked to significant SNPs in Ashanti Dwarf pigs are associated with reduced body weight, smaller litters, and lean meat (ATP8A2, USP12, SPPL2C, PLEKHM1, XXYLT1, ERCC6, BTBD3, SPIDR, and SHH); they are likely to contribute to breed characteristics and were detected in specific populations. Worth mentioning are the THEMIS genes involved with the immune response and regulating T-cell activation; they were identified as being strongly under selection in the same populations [10].

In the central Africa region of Angola, whole-genome sequencing identified selection signatures related to feed efficiency, suggesting that selection towards feed efficiency and metabolism has occurred in local pigs. Of all the genes identified, the CDKAL1 gene, which is associated with insulin and cholesterol metabolism, crucial for feed-utilization efficiency and adaptation traits, was identified as a candidate gene overlapping signatures of selection unique to Angola pigs [13]. These genetic adaptations are likely to enhance the ability of these pigs to utilize low-quality feeds efficiently, supporting their foraging and scavenging abilities [13].

### 3.7. Evidence of Hybridization between Domestic and Wild Pigs and Its Implications

Evidence of gene flow (sharing) between domestic and wild pig populations (warthogs, wild boars, and bush pigs) in these studies is also a phenomenon worth mentioning in this review. It has been reported that the practice of hybridizing domesticated pigs with wild boars has precedent in European farming, where it has been employed to enhance genetic diversity and reproductive performance in commercial pig lines [129]. While strong scientific evidence for hybridization in Africa has remained limited for a long time [9], genetic exchanges are likely to occur in free-range systems where pigs often roam freely, creating opportunities for interaction and potential breeding [12,130]. A study found that genomic sequences of domestic and wild pigs are largely similar, with differences primarily occurring in areas subject to strong selection pressure [131]. Several immune response-related genes, including MHR2, MAP3K12, and NPFF in Kenya [9], and SWAP70 SWAP70, SBF2, FRAS1, NPY2R, and ARHGAP2 in South Africa [8,12], have been notably shared between the domestic pigs and these group of wild suids, indicating a potential flow of genes. Additionally, Mujib et al. reported what is called evidence of the introgression (admixture analysis) of Ugandan bush pigs into domestic pigs for the first time in East Africa. Additionally, Mitochondrial DNA analysis revealed 5–9% wild pig introgression in four pigs initially misidentified as bush pigs, further confirming the evidence of hybridization between domestic and wild pigs [9]. Nonetheless, due to the low genetic diversity observed in bush pigs and warthogs, the study suggests that a more comprehensive genetic analysis using higher-density markers is necessary to confirm this introgression [9]. This evidence of observed hybridization implies that local breeds are likely to confer untapped valuable protective functions that could provide insights into breed-specific disease and climate resistance mechanisms for the case of African swine fever [8,10]. For instance, a follow-up study on the diversity of major histocompatibility complex (MHC) in Kenya found shared SLA-1 and DQB1 alleles between domestic pigs and warthogs, supporting the possibility of viable hybrids between bush pigs and domestic pigs. Specifically, the shared alleles include SLA-108:16 and SLA-114:02 at the SLA-1 locus, SLA-206:07 at the SLA-2 locus, and SLA-DQB107:01:02 at the SLA-DQB1 locus [11]. Thus, leveraging this gene flow between local and wild suids could enhance the resilience and adaptability of local breeds [8]. While hybridization of scavenging pigs and wild suids could contribute to the genetic diversity and adaptive traits observed in village populations, Parkhouse et al. [132] argued that precaution must be made, as these interaction events have also been associated with an outbreak of classical swine fever (CSF) in free-range production systems [132]. Studies suggest that wild suids, such as bush pigs and warthogs, are resistant to ASF and, therefore, serve as potential reservoirs for the virus [131,132]. In Africa, for example, ASF is endemic and circulates through a complex cycle involving wild suids, domestic pigs, and soft ticks of the *Ornithodoros* species [9,131,132]. The main transmission routes to domestic pigs include direct contact with infected wild boars and domestic pigs in free-ranging environments, tick infestation, and the consumption of contaminated feed [9,131,132]. 

## 4. Conclusions and Implications of the Study

This review has presented an overview of African pig genetic resources. Based on a thorough literature review, we determined that African pig genetic resources have the potential to significantly enhance global food security and provide valuable animal proteins due to their genomic regions that help them adapt to harsh environments. These breeds are neglected because of their poor productivity in comparison to commercial breeds. This neglect may result in a decrease in genetic variety, which is harmful in the face of climate change, hence necessitating their sustainable utilization. Specifically, the following key findings are drawn and presented.

- There is a significant gap in the management of African pig genetic resources across the continent, as evidenced by poor, incomplete, and uncoordinated efforts in their characterization.
- Even with existing biases caused by incomplete research and weak genomic tools often being used, existing genomic evidence indicates the following:
  (a) African local pig breeds demonstrate moderate to high levels of genetic diversity, enough to support breeding and conservation programs.
  (b) Low genetic differentiation and evidence of a lack of a distinct population structure suggest that the majority of local pig populations are strained within larger breeds.
  (c) Some early evidence shows selection signatures associated with various adaptive and economic traits, making them good candidates for climate change adaptation strategies and thus ensuring informed breeding programs.
  (d) Reported evidence suggests hybridization and resilience gene exchange among local pigs, wild boars, and warthogs, which could provide untapped insights into breed-specific disease resistance and climate resilience mechanisms. However, a more comprehensive genetic analysis using higher-density markers is warranted to confirm this introgression.
  (e) There is an increasing prevalence of gene introgression from imported commercial breeds across Africa, threatening local adaptation.

### Areas of Future Research and Development

The future research landscape of pig breeding in Africa will increasingly depend on a multi-sectorial approach, integrating genomic advancement technologies and interdisciplinary research. Owing to the low coverage of genome sequencing technology in many of the studies reviewed here, we argue that future research should integrate deeper sequencing technologies that would facilitate the development of SNP bead chips with markers specific to the African pig populations. Additionally, future research should take further steps in using more recent genomic technologies such as exome sequencing, RNA sequencing, and genome-editing technologies like CRISPR to identify, validate, and modify genetic markers underlying some economically important phenotypes seen in local pig populations. These technologies are essential for marker-assisted selection, breed improvement, and conservation efforts. Additionally, to ensure that the unique genetic diversity of local pigs is not lost, deliberate research efforts to preserve these endangered breeds must be increased. The implementation of community-based breeding programs that incorporate both phenotypic and genomic data could help improve the productivity of local breeds at a quicker pace while maintaining their adaptability. Again, social research, such as that concerning the economics of pig rearing in rural environments and its subsequent livelihood contribution to rural households, is also imperative, as it can help convince policymakers and the general public to give more attention to breeding programs focusing on sustainable management. However, to achieve all of these goals, strong emphasis must be placed on stakeholder engagement and the formulation of supportive policies.

## Figures and Tables

**Table 1:** Phenotypic description and performance traits of selected local pig breeds.

Breed	Phenotypic Description	Production and Performance Traits	Reference
Nigerian local pigs	Uniform black/white coat; short, dense hair	▪ Small frame: 49–52 cm height ▪ Slow growth: 19 kg at 6 months, 50 kg at 1 year ▪ Late puberty: 221–302 days ▪ Litter size: 5.3–8.8 piglets ▪ Weaning weight: 4.5–6.2 kg at 90 days Valued marbled meat by consumers	[33,38,53,54,55]
Ashanti Black (Ghana)	Small black pigs with upright ears	▪ Birth weight: 1 kg; ▪ Weaning weight: 5.7–6.2 kg; Mature weight: 25–60 ▪ Litter size: 5–10 piglets ▪ Resilient, heat-tolerant ▪ Perceived disease resistance ▪ Preferred Flavorful meat ▪ Modest feed requirement	[45,48,56,57]
Tswana pigs (Botswana)	Small-framed pigs with solid black or black with white stripes	▪ Litter size: 4.5 piglets litter weight: 25.9 kg ▪ Pre-weaning survival: 96% ▪ Slow growth rate at 0.4 kg/day ▪ Good mothering ability ▪ Highly adaptable to disease and parasites ▪ Ability to use fibrous feeds ▪ Endangered status (pace of population growth is less than one)	[24,58,59]
Kolbroek pigs (South Africa)	Medium-size spotted black and white coat; fat with a short snout, strong feet, and sturdy legs; have a distinct big belly that nearly reaches the ground; origins debated from shipwreck or Portugal	▪ Litter size: 8–10 piglets ▪ Grow faster and attain maturity relatively earlier than other typical local pig breeds ▪ Excellent foragers ▪ Hardy, with good mothering ability	[15,36,60,61,62]
Windsnyer(native to Zimbabwe; a smaller population is found in Mozambique and Zambia)	Windsnyer means “wind-cutter”, derived from its physical appearance characterized by a narrow back with a razorback ridge Small, short, dense hair coats Variable colors: black, brown, white, speckled	▪ Mature female weight: 40–120 kg ▪ Early maturity: 12 weeks ▪ Hardy, disease-resistant ▪ Low carcass quality, owing to their high propensity to deposit back fat thickness ▪ Declining population	[15,46,61,62,63,64]
The Mukota is a domestic pig native to Zimbabwe, with smaller populations in Mozambique and Zambia	Two known types: (1) short, fatty build with a snout resembling a Chinese Lard pig; (2) long snout with razorback resembling the Windsnyer	▪ Uniform black color ▪ Litter size: 6.5–7.5 piglets ▪ Age at first litter: 6–12 months ▪ Heat and disease-tolerant ▪ Less susceptible to *Ascaris suum* ▪ Enlarged caecum—utilizes fiber ▪ Meat is accepted for its organoleptic qualities	[15,40,65,66,67,68]
Ugandan local ecotypes	The majority have a black skin color with long, straight hair, long, thin snouts, and semi-lop ears projecting forward	▪ Average adult body weight: 43.84 ▪ Average body length: 83.91 ▪ Age at first farrowing: 11.62 months ▪ Average litter size: four piglets	[69]
Kenyan local ecotypes (Busia, Kaka mega, Homa Bay)	Varied phenotypes (unclassified)	▪ Mature body weight: 42 kg ▪ Age at first farrowing: 8 months ▪ Weaning age of 5.4 weeks ▪ Litter size: 7.8	[70,71]
Tanzanian local ecotype (Southern highlands)	Varied coat colors, predominantly black, white, and solid black The majority have droopy ears compared to erect ones Long and straight face; short, curled tail	▪ Average mature body weights: boars, 57.4 kg; sows, 54 kg ▪ Mean birth weight: 0.9 kg ▪ Mean weaning weight: 10.8 kg ▪ Average litter size: five to eight piglets ▪ Weaning age of 2.5–3 months ▪ Litter size at weaning: 4.3 piglet	[29,72]

**Table 2:** Genetic diversity indices of Native African pigs.

Region/Country	Breed	Marker	*H_o_*	*H_e_*	F	Reference
West Africa	Nigerian local pigs	12 microsatellites	0.41	0.46	-	[38]
Ghana	Local pigs	Microsatellites	0.469	0.723	0.324	[57]
West Africa	Benin local pigs	17 microsatellites	0.46	0.51	-	[73]
West Africa	Nigeria local	8 microsatellite markers	0.41	0.46		[74]
Southern Africa	Zimbabwe and South Africa	22 microsatellites	0.61–0.75			[15]
Southern Africa	Namibia pigs	39 microsatellites	0.518	0.531	-	[36]
	Mozambique pigs		0.609	0.692		
	Kolbroek pigs		0.537	0.634		
South Africa	Village pigs Village pigs	Porcine SNP60K Porcine SNP60K	0.299	0.359	0.056	[8]
			0.336	0.371	0.198	
	Kolbroek		0.364	0.339	0.051	
	Windsnyer.		0.385	0.360	0.056	
East Africa	Ugandan pigs	Porcine SNP50K	-	-	0.043	[75]
East Africa	Kenya (Homabay)	Porcine SNP60K	0.23	0.64	0.09	
	Kenya (Busia)		0.33	0.88	0.07	[9]
West Africa	Ghanania pigs	Porcine SNP60K	0.28	0.34	0.15	[10]

*H_o_* = observed heterozygosity; *H_e_* = expected heterozygosity; F = inbreeding coefficient.

**Table 3:** Significant SNPs and the associated genes were detected through comparisons between local breeds, wild African pig populations, and commercial breeds.

Breed	SNP	Chr	Gene	Function Description	Reference
South African Village Pig vs. Warthog	rs81355030	1	*RPL18*	Responses to acute heat stress	[96]
	rs81367521	2	*IL17B*	Embryonic development, tissue growth, and inflammatory response	[97]
	rs81285672	12	ARHGAP23	Associated inflammatory response	[98]
	rs81430450	11	*DNAJC15*	Feeding metabolism and efficiency traits	[99]
	rs81430450	11	*EPSTI1*	Variants have shown an association with fertility traits and fat deposition	[100,101]
South African Village vs. Wild Boar	s81244815 2	2	*SWAP70*	Immune cell function, implicated in disease resistance	[102,103]
	rs81244815	2	*SBF2*	Associated with fertility and immune traits	[104]
	rs81401075	8	*FRAS1*	Linked to sow reproduction and feeding traits	[105]
	rs81401075	8	*NPY2R*	Tied to obesity	[106]
South African Village vs. DUR	rs81282695	6	POU3F1	Neurobehavioral functioning	[107]
	rs81282695	6	FHL3	Carcass traits	[108]
South African South African Kolbroek vs. Warthog	rs81341610	3	*LOC102160627*	Unknown function	NA
	rs80993200	4	*ARHGAP39*	Related to milk production and mastitis susceptibility/resistance	[109]
	rs80851822	5	*POLR3B*	Linked to residual feed intake, digestion, and utilization	[110]
	rs80873063	5	*TCP11L2*	Implicated in ovarian follicle development	[111]
	rs80999600	5	*TSPAN9*	Associated with growth rates	[112]
	rs81385003	5	*ITFG2*	Disease resistance	[113]
	rs81285672	12	*ARHGAP23*	Linked to inflammatory response	[99]
	rs81325261	12	*FOXN1*	Important for hair follicle and skin development	[114]
	rs335091311	15	*STAM2*	Residual feed intake	[109]
	rs81453662	15	*DLX1*	Muscling and meat availability	[115]
South African Windsnyer vs. Warthog	rs81381252	4	*ZNF609*	Linked to fertility traits	[110]
	rs81285672	12	*ARHGAP23*	Has been associated with inflammatory pathways	[99]
	rs81325261	12	*FOXN1*	Important for hair follicle and skin development	[116]
	rs331955329	13	*MTMR14*	Implicated in age-related muscle decline	[117]
	rs80971430	13	*BRPF1*	Fatty acid intermuscular profiles	[118]
	rs81248260	13	*ATPB2*	Heat stress on reproductive performance	[119]
	rs80945527	13	*ARPC4*	Muscle weight	[120]
	rs80885182	13	*SEC13*	Muscle weight	[120]
Kenyan population (Busia, Homabay) and Wild African Pig Populations	N/A	5	*MHR2*	Receptor for anti-Mullerian hormone regulates sex differentiation	[9]
	N/A	5	*MAP3K12*	Cell signaling/division	[9]
	N/A	5	*NPFF*	Encode neuropeptide precursor hormone and receptor; modulate pain perception and reproduction	[9]
	N/A	5	*SP1*	Transcription factor binds DNA to regulate gene expression	[9]
	N/A	5	*TARBP2*	Implicated in reproduction and development	[9]
	N/A	5	*U6*	Key RNA component of cellular spliceosome machinery-processing mRNAs	[9]
	N/A	5	*Uc 338*	Was first found to be upregulated in HCC and promote cell growth	[9]
Angola, Iberian, and Large White	N/A	N/A	*NRCAM*	Encodes neural cell adhesion molecules, involved in nervous system development	[13]
Angola and Iberian	N/A	N/A	*SNX2*	Involved in regulating protein trafficking and localization within cells	[13]
Angola and Iberian	N/A	N/A	*RBFOX1*	Influences tissue-specific transcript diversity	[13]
Angola and Iberian	N/A	N/A	*U6*	Key RNA component of cellular spliceosome machinery-processing mRNAs	[13]
Angola and Pietrain, Landrace	N/A	N/A	*SNTG1*	Involved in intracellular transport and cell signaling	[13]
Angola vs. Pietrain	N/A	N/A	*CDKAL1*	Associated with metabolism and feeding efficiency	[13]
Angola and Landrace	N/A	N/A	*DOCK5*	Linked to fat deposition and backfat thickness	[13]
Angola and Landrace	N/A	N/A	*DLGAP2*	Feed intake per feeding	[13]
Kolbroek and Windsyner	N/A	5	*KCNJ3*	Udder structure, signaling pathways	[12]
Kolbroek and Windsyner	N/A	4	*JPH1*	Meat and carcass quality	[12]
South African Village	N/A	NS	*SCPEP1*	Body fat content, intramuscular fat	[12]
South African Village	N/A	12	*PIK3R5*	Litter size, piglets born alive	[12]
South African Village	N/A	2	*TRIM44*	Regulates spinal development	[12]
South African Village	N/A	7	*DPF3*	Influences teat formation	[12]
South African Village	N/A	8	*APBB2*	Modulates fatty acid metabolism	[12]
South African Village	N/A	13	*PTH1R*	Affects limb and skeletal structure	[12]
South African Village	N/A	1	*NEDD4*	Regulates muscle fat deposition	[12]
South African Village	N/A	2	*DMGDH*	Impacts piglet survival at birth	[12]
South African Village	N/A	8	*NR3C2*	Muscle development and teat formation	[12]
South African Village	N/A	12	*A10*	Affects hemoglobin production	[12]
South African Village	N/A	4	*NKAIN3*	Regulates birth weight	[12]
South African Village	N/A	16	*SELENOP*	Affects meat coloration	[12]
South African Village	N/A	16	*PELO*	Influences obesity and teat development	[12]
South African Village	N/A	16	*ITGA2*	Regulates early growth (5 weeks)	[12]
South African Village	N/A	1	*SAMD4*	Modulates intramuscular fat content	[12]
South African Village	N/A	12	*NME1*	Affects various meat quality traits	[12]
South African Village	N/A	12	*CA10*	Influences hemoglobin production	[12]
South African Village	N/A	14	*LIPA*	Regulates multiple growth and carcass traits, wound response, and inflammation	[8,121]
South African Village	N/A	14	*IDE*	Affects various fat deposition traits	[12]
South African Village	N/A	14	*RBP4*	Influences litter size and piglet survival	[12]
South African Village	N/A	14	*EXTL3*	Regulates fat androstenone levels	[12]
South African Village	N/A	8	*FBXW7*	Affects body mass index	[12]
Entire Ghanaian Pig Population	N/A	*-*	*ARID1A*	Linked to abnormal eating behaviors, hyperactivity, decreased body weight, and decreased embryo size	[122]
	N/A	*-*	*ANKFN1*	Linked to abnormal eating behaviors, hyperactivity, decreased body weight, and decreased embryo size	[10,123]
	N/A	11	*ATP8A2*	Plays a role in the regulation of cellular components during cellular organization	[10]
	N/A	*-*	CNTN4	Implicated in abnormal eating behaviors, hyperactivity, decreased body weight, and decreased embryo size	[10,124]

## Data Availability

No new data were created or analyzed in this study.
